# 

**Published:** 1893-09-09

**Authors:** 


**OUR NEW OFFICES,**

428, Strand, W.C., will henceforth be the Office of this Journal.

**Notices of Births, Deaths, and Marriages** are inserted in this column at a charge of 2s. 6d. for an announcement not exceeding four lines.

### NOTICE TO CORRESPONDENTS.

All MS., Letters, Books for Review, and other matters intended for the Editor should be addressed The Editor, The Lodge, Porchester Square, London, W.

The Editor cannot undertake to return rejected MS., even when accompanied by stamped directed envelope.

**Notice to Advertisers and Subscribers.**

All Advertisements, Orders for Copies of The Hospital, and Business Communications should be addressed to The Publisher, *not to the Editor*, The Hospital, 428, Strand, W.C.

The Cost of Subscription, post free, is as follows:—Per week, 2½d.; per quarter, 2s. 6d.; per half-year, 5s.; per annum, 8s. 6d. Foreign:—Per Annum, 10s. 8d.; payable, in all cases, in advance.

**"Burdett's Hospital Annual," 1893.**

Fourth year of publication. 700 pp. Boards, gilt lettering. A most complete guide to British, Colonial, Indian, and American Hospitals, Dispensaries, Nursing and Convalescent Institutions, and Asylums. Price 5s. Published by The Scientific Press (Limited), 428, Strand, W.C.

**NOTICE.—The Index for Vol. XIII. (ending March, 1893) is on sale at the Office of this Journal, price 2½d., post free.**

### MEDICAL VACANCIES AND APPOINTMENTS.

**APPOINTMENTS.**

W. C. Allardice, M.B., C.M.Glasg., appointed Senior House Surgeon to Macclesfield General Infirmary. J. K. Anderson, M.D., L.R.C.S.Edin., reappointed Parochial Medical Officer for St. Vigeans. W. H. Anderson, M.B., C.M.Glasg., appointed Medical Officer for the Third District of the Birkenhead Union. A. J. Bathe, L.R.C.P.Lond., M.R.C.S.Eng., appointed Surgeon to the Southampton Dispensary. H. P. D. A. Benson, appointed Medical Officer for the Skenfrith District of the Monmouth Union. W. B. Betenson, L.R.C.P., M.R.C.S., appointed Medical Officer for the Bungay District of the Wangford Union. G. W. H. Bird, B.A., M.B., B.C.Cantab., L.R.C.P., M.R.C.S., appointed non-Resident House Physician to the St. Thomas's Hospital. J. Bostock, M.R.C.S.Eng., L.S.A., reappointed Medical Officer for the Wymesfold District of the Loughborough Union. H. E. Bower, M.B., C.M.Edin., appointed Medical Officer for the Budworth District of the Runcorn Union. J. W. Boyd, M.B., C.M.Glasg., appointed Junior House Surgeon to the Macclesfield General Infirmary. J. M. Brown, M.B., B.Ch.R.U.I., appointed Medical Attendant to Gilford Mill and Hospital, Surgeon to R.I. Constabulary and Certifying Factory Surgeon. M. A. Cadell, M.D.Brussels, L.R.C.P. and S.Edin., L.F.P.S.Glasg., appointed Medical Officer to the Church of Scotland Mission (Aberdeenshire Branch) at Sialkot, India. R. F. Chance, L.R.C.P., M.R.C.S., appointed Junior Obstetric House Physician to the St. Thomas's Hospital. M. O. Coleman, M.D.Aberd., reappointed Medical Officer and Public Vaccinator for the Surbiton District of the Kingston Union. F. W. H. L. Day, L.R.C.P.Lond., M.R.C.S.Eng., appointed Medical Officer of Health for the Hitchin Rural Sanitary Authority of the Hitchin Union. J. A. Dewar, M.D., L.R.C.S.Edin., reappointed Parochial Medical Officer for the East District of St. Vigeans. W. H. Fawcett, M.D.Brux., D.P.H.Eng., L.R.C.P., L.R.C.S.Ed., L.F.P.S.Glasg., appointed Medical Officer and Public Vaccinator to the Second District of the Wimborne and Langbourne Union and Surgeon to the Workhouse. J. H. Fisher, F.R.C.S., L.R.C.P., appointed Senior Obstetric House Physician to the St. Thomas's Hospital. F. Fowler, L.R.C.P.Lond.,M.R.C.S.Eng., appointed Medical Officer for the Fourth District of the Stroud Union. Dr. Griffin appointed Surgeon to the Southampton Dispensary. E. M. Hainworth, B.Sc.Lond., L.R.C.P., M.R.C.S., appointed Assistant House Surgeon to the St. Thomas's Hospital. T. W. Hicks, L.R.C.P., M.R.C.S., appointed Resident House Physician to the St. Thomas's Hospital. (Extension.) F. D. Holmes, B.A., R.U.I., L.R.C.P.Edin., appointed Medical Officer for the Duffield District of the Belper Union and Medical Officer for the Forth District of the Shardlow Union. C. S. Jaffé, L.R.C.P., M.R.C.S. appointed Clinical Assistant in the special departments for diseases of the throat of the St. Thomas's Hospital. (Extension.) F. Johnson, L.R.C.P.I., M.R.C.S.Eng., reappointed Medical Officer and Public Vaccinnator for the Hedgerly Sanitary District of the Eton Union. F. Johnson M.B.Lond., F.R.C.S.Eng., appointed Senior House Surgeon to the Deaconess's Institution and Hospital, Tottenham, N. A. W. Leachman, M.D.St.And., M.R.C.S.Eng., appointed Medical Officer of Health for the Petersfield Urban Sanitary District. J. W. C. Merry, M.A., M.B., B.Ch.Oxon., appointed Clinical Assistant in the special departments for diseases of the skin of the St. Thomas's Hospital. A. R. O. Milton, L.R.C.P., M.R.C.S., appointed Assistant House Surgeon to the St. Thomas's Hospital. H. M. Moore, L.R.C.P., M.R.C.S., appointed Clinical Assistant in the special departments for Diseases of the Ear of the St. Thomas's Hospital. John D. S. Nodes, L.R.C.P.Lond., M.R.C.S. Eng., appointed Medical Officer for the Second District of the

East Preston Union. P. Northcote, L.R.C.P., M.R.C.S., appointed Non-Resident House Physician to the St. Thomas's Hospital. (Extension.) F. Pershouse, L.R.C.P., M.R.C.S., appointed Clinical Assistant in the special departments for Diseases of the Skin of the St. Thomas's Hospital. (Extension.) C. P. Planck, M.A.Cantab., L.R.C.P., M.R.C.S., appointed House Surgeon to the St. Thomas's Hospital. W. P. Purvis, M.B., B.Sc.Lond., L.R.C.P., M.R.C.S., appointed Clinical Assistant in the special departments for Diseases of the Throat of St. Thomas's Hospital. W. Redpath, L.R.C.P., M.R.C.S., appointed House Surgeon to the St. Thomas's Hospital. B. M. H. Rogers, M.D., B.Ch., M.R.C.S., appointed Physician to the Bristol Hospital for Sick Children and Women. E. Smith, L.R.C.P., M.R.C.S., appointed House Surgeon to the St. Thomas's Hospital. Mr. W. M. Steinthal, appointed Assistant Medical Officer of Health to the Manchester Union. G. W. Thompson, B.A., M.B., B.C. Cantab., L.R.C.P., M.R.C.S., appointed Resident House Physician to the St. Thomas's Hospital. L. C. T. Thorne, M.R.C.S., L.R.C.P., appointed Assistant House Surgeon to the Infirmary for Children, Liverpool. Mr. R. T. Turner, appointed Assistant Medical Officer to the Manchester Union. F. K. Tweedie, M.B.R.U.I., B.Ch., appointed Medical Officer of Health for the Hucknall-under-Huthwaite Urban Sanitary District of the Mansfield Union. C. S. Wallace, F.R.C.S., L.R.C.P., appointed House Surgeon to the St. Thomas's Hospital. M. A. Ward, M.D.Dublin., F.R.C.S.I., appointed Medical Officer of Health for Rathmines. G. G. Whitwell, M.B., C.M Edin., appointed Medical Officer for the Atcham District of the Atcham Union. A. Wilsden, C.M., M.B.Aberd., appointed Junior House Surgeon to the Deaconess's Institution and Hospital, Tottenham, N. A. H. Woodcock, L.R.C.P., M.R.C.S., appointed Clinical Assistant in the special departments for Diseases of the Ear of the St. Thomas's Hospital. (Extension.) Mr. W. Griffith, M.B., Ch.B. Victoria University (late of the Manchester Southern Hospital and the Denbighshire Infirmary), has been appointed House Surgeon to the London Temperance Hospital. Mr. G. Templeton, M.B., C.M.Edin., L.R.C.P.Lond., M.R.C.S.Eng., has been appointed Junior House Surgeon to the London Temperance Hospital.

### VACANCIES.

The following vacancies are announced this week, the amount of salary in each case being given after the name of the institution:

### Resident Medical Officers.

Camberwell House. Junior Assistant Medical Officer, unmarried. Initial salary £100, with subsequent increase, board, lodging, and washing. Applications to the Medical Superintendent, 33, Peckham Road, S.E.

City of London Hospital for Diseases of the Chest, Victoria Park, E. House Physician. Appointment for six months. Board, residence, and allowance for washing provided. Applications to the Secretary, at the Office, 24, Finsbury Circus, E.C., by September 14th.

Denbighshire Infirmary, Denbigh. House Surgeon; must be conversant with the Welsh language. Salary to commence £85 per annum, with board, residence, and washing (at the end of year's engagement, if satisfactory, a gratuity of £15 is given). Applications to W. Vaughan Jones, Secretary.

Kent County Asylum, Chartham, near Canterbury. Junior Assistant Medical Officer, unmarried. Salary £125 per annum, with furnished apartments, board, and attendance. Applications to the Clerk of the Committee of Visitors, Allen Fielding, Solicitor, Canterbury, by September 20th.

St. Peter's Hospital for Stone and Urinary Diseases, Henrietta Street, Covent Garden. House Surgeon; must be M.R.C.S.; appointment for six months. Honorarium 25 guineas, with board, lodging, and washing. Applications to the Secretary by September 11th.

Stafford General Infirmary, Stafford. Assistant House Surgeon. Board and residence provided. Applications to the Secretary by September 9th.

West London Hospital, Hammersmith Road, W. House Physician and House Surgeon. Appointment for six months. Board and lodging provided. Must be registered under the Medical Act. Applications, with testimonials, must be sent to R. J. Gilbert, Secretary-Superintendent, by September 20th.

### Non-Resident Medical Officers.

University of Aberdeen. Eleven Examiners in Medicine. The Examiners in Medicine and Surgery will receive a grant of £50 each, and the other nine a grant of £40 each. Applications to Robert Walker, Secretary, the University Court, by September 25th.

University of Durham. Professor of Comparative Pathology in the University of Durham College of Medicine, Newcastle-on-Tyne. Applications to the trustees of the late Dr. G. Y. Heath, care of Dr. Philipson, 7, Eldon Square, Newcastle-on-Tyne, by September 9th.

